# Retinal and choroidal thickness in fuchs uveitis syndrome: a contralateral eye study

**DOI:** 10.1186/s12886-024-03554-y

**Published:** 2024-07-12

**Authors:** Farzan Kianersi, Athar Taheri, Majid Mirmohammadkhani, Mohammadreza Akhlaghi, Alireza Peyman, Hamidreza Kianersi, Matin Irajpour, Hanieh Kianersi, Mohsen Pourazizi

**Affiliations:** 1 Isfahan Eye Research Center, Department of Ophthalmology, Isfahan University of Medical Sciences, Isfahan, Iran; 2 Social Determinants of Health Research Center, Semnan University of Medical Sciences, Semnan, Iran; 3 Department of Epidemiology and Biostatistics, School of Medicine, Semnan University of Medical Sciences, Semnan, Iran; 4 Student Research Committee, Isfahan University of Medical Sciences, Isfahan, Iran

**Keywords:** Fuchs Uveitis Syndrome, Fuchs Heterochromic Iridocyclitis, Anterior uveitis, Choroidal Thickness, Choriocapillary, Central macular thickness, Central macular volume

## Abstract

**Background:**

To investigate the subfoveal retinal and choroidal thickness in patients with unilateral Fuchs Uveitis Syndrome (FUS).

**Methods:**

This comparative contralateral study was performed in affected eyes with FUS versus fellow eyes. For each eye parameters such as subfoveal choroidal thickness (SCT), subfoveal choriocapillary thickness (SCCT), central macular thickness (CMT), and central macular volume (CMV) were measured; then the measured values of affected and fellow unaffected eye were compared.

**Results:**

Thirty-seven patients (74 eyes) including 19 females (51.4%) with a mean age of 36.9 ± 7.6 years were enrolled. The mean SCT was lower in the affected eyes (344.51 ± 91.67) than in the fellow (375.59 ± 87.33) with adjusting for duration of disease and axial lengths (*P* < 0.001). The mean SCCT, CMT, and CMV were higher in eyes with FUS than in fellow eyes (*P* < 0.05).

**Conclusions:**

The result of our study demonstrated that affected eyes in patients with FUS tend to have thinner SCT and thicker SCCT and CMT compared to uninvolved fellow eyes.

## Background

Fuchs uveitis syndrome (FUS), also known as Fuchs Heterochromic Iridocyclitis (FHI), is typically a unilateral slow progressive chronic anterior uveitis characterized by white, diffuse stellate keratic precipitates (KP) on the corneal endothelium. Other characteristics of this condition are as follows: mild anterior chamber reaction, lack of posterior synechia, anterior vitreous disorders, and iris atrophy with or without iris heterochromia [1–4]. International Uveitis Study Group defines FUS as a nongranulomatous inflammation with insidious onset and low grade activity [5]. This condition is mostly seen in third and fourth decades of life. Studies from different ethnic backgrounds have demonstrated that different races have various clinical manifestations of FUS [5].

Although the exact etiopathogenesis of FUS is unclear, there are several theories about underlying pathogenesis of FUS, such as: genetic susceptibility, immune-mediated, sympathetic, vascular and infectious theories [6–11]. Also, associations with several post-infectious conditions such as toxoplasma and rubella have been proposed [12–14]. Thus, FUS is probably a secondary condition to a variety of insults with a spectrum of clinical signs and multiple etiologic factors.

FUS is considered a low-grade inflammation in the anterior chamber (anterior uveitis); however, a limited number of studies have shown evidence for posterior segment involvement as well [15–18]. Enhanced depth imaging optical coherence tomography (EDI-OCT) has been used to document the changes in choroidal thickness in several uveitis conditions [19–22]. Regarding choroidal thinning, there are conflicting evidence and no clear consensus has been reached [23–25]. One study has found no evidence of retinal thinning in FUS [26].

So, the present study aimed to evaluate the choroidal and retinal thickness in the subfoveal area in patients with unilateral FUS and compare the measurements with their unaffected fellow eye. One major limitation of prior studies that we aimed to address in this study was to negate the probable effects of disease duration on thickness measurements.

## Materials and methods

### Participant

This comparative contralateral study was performed in the uveitis outpatient clinic of Feiz Ophthalmology Hospital (a referral center for eye disorders) affiliated with the Isfahan University of Medical Sciences (IUMS), Isfahan, Iran, between November 2020 and September 2022. The protocol of this study was approved by the Institutional Ethics Committee of IUMS, Isfahan, Iran (IR.MUI.MED.REC.1397.307). After an adequate explanation of the project to the patients, a written informed consent was obtained before enrollment to the study. Consecutive cases of unilateral FUS aged above 18 years, after a comprehensive ocular and systemic evaluation were included in the study.

Patients with pregnancy or lactation; bilateral FUS cases; monoocularity; history of the retinal or macular abnormalities; history of ocular trauma; history of any previous laser photocoagulation and/or intraocular surgery (except cataract surgery), use of any systemic medications during 6 months prior to investigations and current or previous posterior segment diseases such as toxoplasmosis scar, cystoid macular edema (CME) and choroidal neovascularization (CNV) were excluded.

The diagnosis of FUS was established based on clinical signs including keratic precipitates, iris atrophy with or without heterochromia, mild flare and minimal cells in the anterior chamber (AC), vitreous involvement, and absence of posterior synechiae or CME [1, 27].

This report follows Strengthening the reporting of observational studies in epidemiology (STROBE) guideline for case-control studies.

### Study protocols

In the baseline examinations, the patients underwent complete ophthalmologic examinations, including best-corrected visual acuity (BCVA) measurement using a Snellen chart, Goldmann applanation tonometry, Axial length (AL) measurement by swept-source biometry (ZEISS IOL Master 700, Carl Zeiss Meditec AG, Jena, Germany), anterior segment slit-lamp and dilated biomicroscopic fundoscopy with 90 D lens. Also, data such as patients’ age, gender, duration of disease, ocular, and past medical history were recorded along with more detailed ophthalmic examination (Iris atrophy, presence of opacities in the crystalline lens, and glaucoma), and their optical coherence tomography (OCT) parameters. Systemic evaluation and laboratory investigations were performed for the purpose of ruling out another differential diagnoses.

Macular and choroidal thickness were measured using OCT (Heidelberg Engineering, Heidelberg, Germany). Enhanced depth imaging OCT (EDI-OCT) imaging was obtained using The EDI mode of the same OCT device that was described previously [28].

The thickness of the 1-mm central retina is referred to as central macular thickness (CMT) and the central macular volume (CMV) is determined as reported in the OCT prints. Subfoveal choroidal thickness (SCT) was defined as the vertical distance from the hyperreflective line of Bruch’s membrane to the hyperreflective line of the inner surface of the sclera [25]. Subfoveal choriocapillary thickness (SCCT) was considered from the hyperreflective line of Bruch’s membrane to the settler’s layer. All measurement was obtained by the same ophthalmologists with the manual caliper tool of the OCT software, and the average of two measurements was recorded for analysis.

In this assessment, the outcome was defined by the differences observed in the SCT, SCCT, CMT, and CMV between the eyes affected by FUS and the fellow eyes.

### Statistical analysis methods

Mean and standard deviation (SD) of age and duration of disease (year) were reported for each patient, as well as for AL, BCVA, and IOP, for each eye (affected and fellow). For categorical variables such as sex, side of involvement, history of cataract surgery, and iris atrophy, frequency distribution was reported in terms of count and percentage. The dependent variables such as CMT, CMV, SCT, and SCCT, were compared between subgroups of categorical variables using T-Test assuming if there was a normal distribution; otherwise, Mann-Whitney Test was used. Considering the mentioned assumption, Paired Samples T-Test or Wilcoxon Signed Ranks Test were used to compare these values ​​between the two eyes (affected vs. fellow). The normality of the distributions was assessed using Shapiro-Wilk Test. The relationship between each of the dependent and the numerical variables of the study, namely age, AL, duration of disease, BCVA, and IOP, were explored using the Spearman correlation coefficient. Comparison of the two eyes (affected vs. fellow) for each of the response variables was performed using the Pillai’s Trace Multivariate Test of General Linear Model, adjusting for AL and duration of disease and dependency between the two eyes in each participant. The software used was SPSS-18 (Statistical Package for Social Science, IBM- SPSS Inc, Chicago, USA). *P* values ​​less than 0.05 were considered significant in all tests. According to study conducted by Balci and Ozsutc [25] and using G*power software, minimum sample size of 30 was calculated for this study.

## Results

Thirty-seven patients (74 eyes) including 19 females (51.4%) with a mean age of 36.9 ± 7.6 years were enrolled in this study. The mean (± SD) duration of the disease was 6.1 ± 3.6 years. The mean AL was 23.67 ± 1.44 in eyes with FUS and 23.43 ± 0.87 in eyes without FUS. Table 1 presents patients’ demographics and clinical findings (Table 1).

**Table 1:** Demographic and clinical findings of patients with FUS who enrolled in the study

Characteristics	Subgroups	Mean ± SD/Count (%)
Age (year)		36.9 ± 7.6
Duration of disease (year)		6.1 ± 3.6
Sex	Male	18 (48.6)
	Female	19 (51.4)
Laterality of affected eye	Right	14 (37.8)
	Left	23 (62.2)
AL	Affected eye	23.67 ± 1.44
	Fellow eye	23.43 ± 0.87
LogMAR BCVA	Affected eye	0.56 ± 0.52
	Fellow eye	0.15 ± 0.14
IOP (mmHg)	Affected eye	15.76 ± 4.22
	Fellow eye	14.57 ± 2.21
Anterior chamber cells grading	0.5+	12 (32.43)
	1+	13 (35.14)
	2+	12 (32.43)
Vitreous cells grading	No cell	12 (32.43)
	0.5+	11 (29.73)
	1+	14 (37.84)
Iris atrophy	Yes	37 (100)
History of cataract surgery	No	26 (70.3)
	Yes	11 (29.7)

AL: axial lengths; BCVA: best-corrected visual acuity; IOP: intraocular pressure; SD: standard deviation

**Fig. 1:**
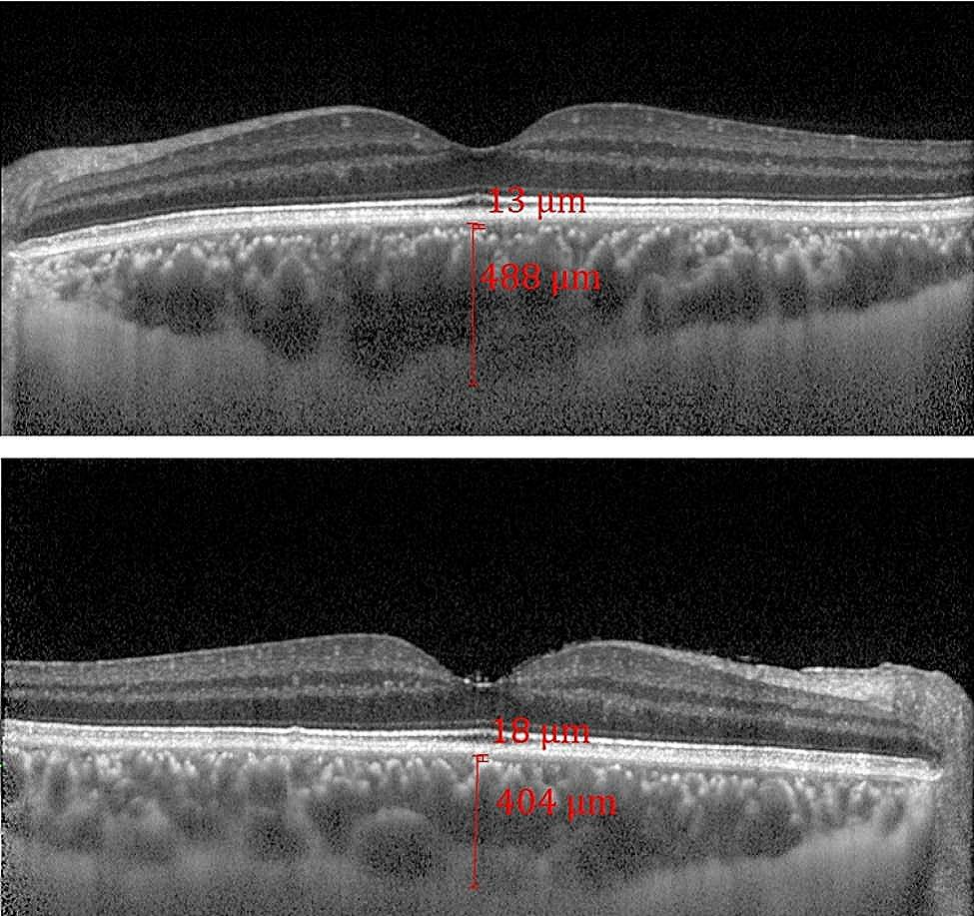
Representative EDI-OCT images of the choroid of a patient with FUS. (a) subfoveal choroidal thickness (SCT) and subfoveal choriocapillary thickness (SCCT) of the affected eye in a patient with FUS. (b) SCT and SCCT of the fellow eye of the same patient with FUS.

The mean SCT was lower in eyes with FUS (344.51 ± 91.67) than in fellow eyes (375.59 ± 87.33) without (*P* < 0.014) and with adjusting (*P* < 0.001) for the duration of disease and AL (Fig. 1). The mean SCCT was found to be 32.35 ± 13.11 μm in eyes with FUS and 28.05 ± 8.32 μm in eyes without FUS. A significant difference was found in SCCT between groups only after adjusting for duration of disease and AL (*P* = 0.012). Significant differences were observed in CMT (*P* < 0.001) and CMV (*P* < 0.001) between the eyes with FUS and fellow eyes, regardless of adjustment for duration of disease and AL. The mean CMT and CMV were higher in eyes with FUS than in fellow eyes without and with adjusting (Table 2). The results of CMT, CMV, SCT and SCCT obtained by SD-OCT are summarized in Table 2.

**Table 2:** Comparison of CMT, CMV, SCT, and SCCT between the two eyes (affected vs. fellow) with and without adjusting for duration of disease and AL

Measurement	Mean ± SD		*P*	*P* ^*^
	Affected eye	Fellow eye		
**CMT**	274.22 ± 25.93	262.51 ± 27.17	0.001^†^	< 0.001
**CMV**	8.79 ± 0.55	8.61 ± 0.47	< 0.001^†^	< 0.001
**SCT**	344.51 ± 91.67	375.59 ± 87.33	0.014^**^	< 0.001
**SCCT**	32.35 ± 13.11	28.05 ±8.32	0.112^†^	0.012

*Pillai’s Trace Multivariate test of General Linear Model adjusting for duration of disease and AL, **Paired Samples T-Test, †Wilcoxon Signed Ranks Test

AL: axial lengths; CMT: central macular thickness; CMV: central macular volume; SCT: subfoveal choroidal thickness; SCCT: subfoveal choriocapillary thickness

Correlation analysis was performed between characteristics of affected eyes with FUS and CMT, CMV, SCT, and SCCT (Table 3). There was negative correlation between LogMAR BCVA and CMT (*r*=-0.40, *P* = 0.01) as well as the CMV (*r*=-0.31, *p* = 0.05). There was no statistically significant correlation between other characteristics of affected eyes, such as degree of inflammation in anterior chamber and vitreous, duration of disease and axial length, with FUS and CMT, CMV, SCT, and SCCT (*P* > 0.05 for all correlations).

**Table 3:** Correlation between characteristics of affected eyes with FUS and CMT, CMV, SCT, and SCCT

Characteristics	Correlation^*^ (*P*)				
	CMT	CMV	SCT	SCCT	
**Age** (year)	-0.006 (0.97)	-0.081(0.63)	0.041(0.810)	-0.072(0.67)	
**AL of the affected eye** (mm)	-0.033 (0.84)	-0.222(0.18)	0.077(0.651)	-0.149(0.38)	
**Duration of disease** (year)	-0.061 (0.72)	-0.071(0.67)	-0.145(0.39)	0.098(0.56)	
**BCVA** (LogMAR)	-0.401(0.01)	-0.315(0.05)	0.080(0.63)	0.186(0.27)	
**IOP** (mmHg)	-0.133 (0.43)	-0.293(0.07)	0.071(0.67)	-0.125(0.46)	

^*^Spearman Correlation Coefficient

AL: axial lengths; BCVA: best-corrected visual acuity; IOP: intraocular pressure; CMT: central macular thickness; CMV: central macular volume; SCT: subfoveal choroidal thickness; SCCT: subfoveal choriocapillary thickness

## Discussion

The results of our study demonstrated two changes in eyes with FUS while comparing with their fellow unaffected eyes: subfoveal choroidal thinning and subfoveal choriocapillary thickening. Also, the mean CMT and CMV were higher in eyes with FUS than in fellow. The importance of present study is accounting for disease duration, in comparing the measurements from affected and fellow eyes of FUS patients; this was a major limitation that was present in all of previous studies. Given the progressive nature of FUS this limitation severely hampers the interpretability of findings in prior investigations. Noting that FUS progresses insidiously, and patients only become symptomatic after experiencing floaters or decreased vision (due to cataracts), it’s reasonable to assume that the actual duration of the disease is often underestimated. Although, our findings did suggest a negative correlation between duration of disease and different measurements, they were not statistically significant, which could be attributable to small sample size.

Thinner SCT and thicker SCCT in affected eyes with FUS could be attributed to the difference in structural properties of choroidal layers. The choriocapillaris is a continuous layer of large capillaries lying in a single plane beneath the retinal pigment epithelium (RPE) [29]. The vessel walls are extremely thin and contain multiple fenestrations, especially on the surface facing the retina. In contrast, the middle and outer layers of choroidal vessels are not fenestrated [30]. In the acute phase of inflammation these fenestrations can cause increased choroidal blood flow, leading to choriocapillaris thickening [29, 31, 32].

The current studies regarding retinal and choroidal thickness in FUS are very limited and in some cases their findings conflict each other [23–26, 33]. Kardes et al. demonstrated that mean ganglion cell complex thickness and subfoveal choroidal thickness in the affected eyes of patients with FUS are reduced compared to the unaffected eyes, whereas the retinal nerve fiber layer (RNFL) thickness and macular thickness were not significantly different between eyes [23].

In a study by Cerquaglia A, choroidal thickness of sixteen eyes of eight consecutive patients with unilateral FUS was measured using EDI-OCT. Their findings demonstrated significant full-thickness choroidal thinning compared to the unaffected eye [24]. This study did not consider disease duration as a confounding factor.

In another study, Ozlem Balci et al. compared RNFL, macular and choroidal thickness between the affected and unaffected eyes of FUS patients, and the eyes of healthy control subjects matched with patients. They found choroidal thinning at the fovea and each point within the horizontal nasal and temporal quadrants in the affected eyes of FUS compared with the unaffected eyes or the eyes of control subjects; whereas there were no statistically significant difference in RNFL and macular thickness values [25]. In an update to their 2016 study, Balci et al. published another study in 2022 [26]; in which they observed no statistically significant difference while comparing central choroidal and retinal thickness of eyes affected by FUS and fellow unaffected eyes and healthy controls. However, once again they did not take disease duration into account.

Przybyś et al. have conducted a similar study, in which subfoveal choroidal thickness was measured and compare with fellow unaffected eyes as well as a healthy control group. Their findings revealed a significant intereye difference in SCT of FUS patients compared to healthy controls. But no statistically significant difference was observed when comparing the different measurements of eye groups (affected and unaffected eyes of FUS patients, left and right eye of healthy controls) [33].

Although previous studies mainly focused on the inflammatory process and diagnostic criteria of FUS, all of them as well as the present study proposed the involvement of the ocular posterior segment (e.g. vitreous) in FUS; manifesting as thinning of choroidal layer, probably due to a diffuse inflammatory reaction.

The acute and chronic inflammatory processes can lead to different effects on choroids. In the acute phase, an increase in choroidal thickness in inflammatory uveitis (e.g. Vogt-Koyanagi-Harada disease) can be correlated with anterior chamber inflammation score [34–36]. This increase in thickness of the choroid could be attributed to the increased blood flow during the acute phase of inflammation; hence choroidal effusion is the mechanism responsible for choroidal thickening in this phase [37].

In contrast, the possible explanation of choroidal thinning in long-lasting disease can be attributed to chronic inflammation and ischemic changes caused by recurrent inflammation; that may lead to fibrosis formation and decrease the subfoveal thickness. So subfoveal thinning in FUS can be related to long-standing disease course [20, 24, 38].

Additionally, we found the mean CMT and CMV were higher in eyes with FUS than in fellow eyes. Glaucoma and ocular hypertension have been reported as common complications of FUS in several reports [39]. In the present study, we did not include eyes with glaucoma and ocular hypertension. Eleven of our patients (29.7%) had already undergone cataract surgery in the affected eye, because of severity of unilateral cataract caused by FUS. Several studies have already reported how cataract surgery and inflammation can induce a long-term increase in CMT [40, 41]. Considering this and the fact that FUS patients have recurrent inflammation, we can explain the observed increase in CMT of affected eyes.

We are aware of the limitations of the present study. One limitation of the current study was that it included a sample of unilateral FUS without healthy subjects. Another limitation of our study was the lack of measurement of choroidal thickness in multiple sites. Also, a noteworthy limitation in this study is underestimating the duration of FUS, which is caused by the initial asymptomatic period of this disease. This work could be a basis for next, prospective studies for evaluating OCT measurements with duration of disease, recurrence and severity of FUS; which could turn into a useful tool for clinicians.

## Conclusions

In conclusion, after adjusting for the duration of disease, compared to the uninvolved fellow eyes we observed subfoveal choroidal thinning in eyes with FUS. Also increased CMT and CMV was noted in affected eyes, regardless of adjustment for duration of disease. To provide better insights into structural changes of the choroid in FUS, future investigations should measure choroidal and retinal thickness in multiple sites with large sample sizes both in acute and chronic phases.

## Data Availability

The data supporting the findings of this study are accessible upon request from the corresponding author.

## Abbreviations

FUS: Fuchs Uveitis Syndrome
FHI: Fuchs Heterochromic Iridocyclitis
SCT: Subfoveal choroidal thickness
SCCT: Subfoveal choriocapillary thickness
CMT: Central macular thickness
CMV: Central macular volume
KP: Keratic precipitates
EDI-OCT: Enhanced depth imaging optical coherence tomography
CME: Cystoid macular edema
CNV: Choroidal neovascularization
AC: The anterior chamber
BCVA: Best-corrected visual acuity
AL: Axial length
RNFL: Retinal nerve fiber layer
RPE: Retinal pigment epithelium

## References

[1] Kimura SJ, Hogan MJ, Thygeson P (1955). Fuchs’ syndrome of heterochromic cyclitis. AMA Arch Ophthalmol.

[2] Abano JM, Galvante PR, Siopongco P, Dans K, Lopez J (2017). Review of epidemiology of Uveitis in Asia: pattern of Uveitis in a Tertiary Hospital in the Philippines. Ocul Immunol Inflamm.

[3] Ozcelik-Kose A, Balci S, Turan-Vural E (2021). In vivo analysis of choroidal vascularity index changes in eyes with Fuchs Uveitis syndrome. Photodiagn Photodyn Ther.

[4] Nasrollahi K, Fazel F, Mirjani T, Kianersi F, Fazel M, Pourazizi M (2022). Ultra-widefield Fundus Fluorescein Angiography findings in patients with Fuchs’ Uveitis Syndrome. Adv Biomed Res.

[5] Sun Y, Ji Y (2020). A literature review on Fuchs Uveitis syndrome: an update. Surv Ophthalmol.

[6] La Hey E, de Jong PT, Kijlstra A (1994). Fuchs’ heterochromic cyclitis: review of the literature on the pathogenetic mechanisms. Br J Ophthalmol.

[7] Ganesh SK, Sharma S, Narayana KM, Biswas J (2004). Fuchs’ heterochromic iridocyclitis following bilateral ocular toxoplasmosis. Ocul Immunol Inflamm.

[8] Stunf S, Petrovec M, Zigon N, Hawlina M, Kraut A, de Groot-Mijnes JD, Valentincic NV (2012). High concordance of intraocular antibody synthesis against the rubella virus and Fuchs heterochromic uveitis syndrome in Slovenia. Mol Vis.

[9] Li H, Hou S, Du L, Zhou Q, Kijlstra A, Liu Q (2014). Polymorphisms of TNFAIP3 gene in a Chinese Han population with fuchs heterochromic iridocyclitis. Ophthalmic Genet.

[10] Kianersi F, Mohammadi Z, Ghanbari H, Ghoreyshi SM, Karimzadeh H, Soheilian M (2015). Clinical patterns of Uveitis in an Iranian Tertiary Eye-Care Center. Ocul Immunol Inflamm.

[11] Suzuki J, Goto H, Komase K, Abo H, Fujii K, Otsuki N, Okamoto K (2010). Rubella virus as a possible etiological agent of Fuchs heterochromic iridocyclitis. Graefe’s Archive Clin Experimental Ophthalmol.

[12] de Groot-Mijnes JD, de Visser L, Rothova A, Schuller M, van Loon AM, Weersink AJ (2006). Rubella virus is associated with fuchs heterochromic iridocyclitis. Am J Ophthalmol.

[13] Accorinti M, Gilardi M, Pirraglia MP, Amorelli GM, Nardella C, Abicca I, Pesci FR (2014). Cytomegalovirus anterior uveitis: long-term follow-up of immunocompetent patients. Graefes Arch Clin Exp Ophthalmol.

[14] Toledo de Abreu M, Belfort R, Hirata PS (1982). Fuchs’ heterochromic cyclitis and ocular toxoplasmosis. Am J Ophthalmol.

[15] Bouchenaki N, Herbort CP (2010). Fluorescein angiographic findings and clinical features in Fuchs’ uveitis. Int Ophthalmol.

[16] Bouchenaki N, Herbort CP (2009). Fuchs’ Uveitis: failure to associate Vitritis and Disc Hyperfluorescence with the Disease is the Major Factor for Misdiagnosis and Diagnostic Delay. Middle East Afr J Ophthalmol.

[17] Kianersi F, Kianersi H, Pourazizi M, Beni AN, Noorshargh P (2024). Fuchs’ uveitis syndrome: a 20-year experience in 466 patients. Sci Rep.

[18] Kianersi F, Mortazavi SA, Peyman A, Rahimi F, Pourazizi M. Ultrasound biomicroscopic findings in Fuchs Uveitis syndrome: a contralateral eye study. Saudi J Ophthalmol. 2024. 10.4103/sjopt.sjopt_166_23 PMC12685257 41367835

[19] Güngör SG, Akkoyun I, Reyhan NH, Yeşilırmak N, Yılmaz G (2014). Choroidal thickness in ocular sarcoidosis during quiescent phase using enhanced depth imaging optical coherence tomography. Ocul Immunol Inflamm.

[20] Coskun E, Gurler B, Pehlivan Y, Kisacik B, Okumus S, Yayuspayi R (2013). Enhanced depth imaging optical coherence tomography findings in Behcet disease. Ocul Immunol Inflamm.

[21] Kianersi F, Rezaeian-Ramsheh A, Pourazizi M, Kianersi H (2018). Intravitreal diclofenac for treatment of refractory uveitis-associated cystoid macular oedema: a before and after clinical study. Acta Ophthalmol.

[22] Kianersi F, Rezaeian-Ramsheh A, Rahimi A, Akhlaghi M, Dehghani A, Farajzadegan Z, Pourazizi M. Non-steroidal intravitreal injection for noninfectious uveitic cystoid macular edema: systematic review and meta-analysis. Eur J Ophthalmol. 2023:11206721231212777. 10.1177/11206721231212777 37933173

[23] Kardes E, Sezgin Akcay BI, Unlu C, Ergin A (2017). Choroidal thickness in eyes with Fuchs Uveitis Syndrome. Ocul Immunol Inflamm.

[24] Cerquaglia A, Iaccheri B, Fiore T, Lupidi M, Torroni G, Fruttini D (2016). Full-thickness choroidal thinning as a feature of Fuchs Uveitis Syndrome: quantitative evaluation of the choroid by enhanced depth imaging Optical Coherence Tomography in a cohort of consecutive patients. Graefes Arch Clin Exp Ophthalmol.

[25] Balci O, Ozsutcu M (2016). Evaluation of retinal and choroidal thickness in Fuchs’ Uveitis Syndrome. J Ophthalmol.

[26] Balci AS, Pehlivanoglu S, Basarir B, Altan C (2023). Comparison of retinal and choroidal changes in Fuchs’ uveitis syndrome. Int Ophthalmol.

[27] Mohamed Q, Zamir E (2005). Update on Fuchs’ uveitis syndrome. Curr Opin Ophthalmol.

[28] Spaide RF, Koizumi H, Pozzoni MC (2008). Enhanced depth imaging spectral-domain optical coherence tomography. Am J Ophthalmol.

[29] Lejoyeux R, Benillouche J, Ong J, Errera M-H, Rossi EA, Singh SR (2022). Choriocapillaris: fundamentals and advancements. Prog Retin Eye Res.

[30] Singh SR, Vupparaboina KK, Goud A, Dansingani KK, Chhablani J (2019). Choroidal imaging biomarkers. Surv Ophthalmol.

[31] Gabriel M, Kruger R, Shams-Mafi F, Hermann B, Zabihian B, Schmetterer L (2017). Mapping retinal and Choroidal Thickness in Unilateral Nongranulomatous Acute Anterior Uveitis using three-dimensional 1060-nm Optical Coherence Tomography. Invest Opthalmology Visual Sci.

[32] Invernizzi A, Cozzi M, Staurenghi G (2019). Optical coherence tomography and optical coherence tomography angiography in uveitis: a review. Clin Exp Ophthalmol.

[33] Przybyś M, Brydak-Godowska J, Kęcik D (2021). Subfoveal Choroidal Thickness and its Intereye differences in Fuchs Uveitis Syndrome evaluated using Optical Coherent Tomography. Ocul Immunol Inflamm.

[34] Nakayama M, Keino H, Okada AA, Watanabe T, Taki W, Inoue M, Hirakata A (2012). Enhanced depth imaging optical coherence tomography of the choroid in Vogt-Koyanagi-Harada disease. Retina.

[35] Maruko I, Iida T, Sugano Y, Oyamada H, Sekiryu T, Fujiwara T, Spaide RF (2011). Subfoveal choroidal thickness after treatment of Vogt-Koyanagi-Harada disease. Retina.

[36] Ishikawa S, Taguchi M, Muraoka T, Sakurai Y, Kanda T, Takeuchi M (2014). Changes in subfoveal choroidal thickness associated with uveitis activity in patients with Behcet’s disease. Br J Ophthalmol.

[37] Akcar N, Goktekin F, Ozer A, Korkmaz C (2010). Doppler sonography of ocular and carotid arteries in Behcet patients. J Clin Ultrasound.

[38] Maneschg OA, Volek E, Nemeth J, Somfai GM, Gehl Z, Szalai I, Resch MD (2014). Spectral domain optical coherence tomography in patients after successful management of postoperative endophthalmitis following cataract surgery by pars plana vitrectomy. BMC Ophthalmol.

[39] Babu K, Adiga M, Govekar SR, Kumar BR, Murthy KR (2013). Associations of Fuchs heterochromic iridocyclitis in a south Indian patient population. J Ophthalmic Inflamm Infect.

[40] Yilmaz T, Karci AA, Yilmaz I, Yilmaz A, Yildirim Y, Sakalar YB (2016). Long-term changes in Subfoveal Choroidal Thickness after cataract surgery. Med Sci Monit.

[41] Noda Y, Ogawa A, Toyama T, Ueta T (2014). Long-term increase in subfoveal choroidal thickness after surgery for senile cataracts. Am J Ophthalmol.

